# Reviewer acknowledgement

**DOI:** 10.1186/s13028-016-0195-6

**Published:** 2016-02-16

The *Acta Veterinaria Scandinavica* editorial team would sincerely like to thank all of our reviewers who contributed to peer review for the journal in 2015.

**Argentina**

Rodolfo Luzbel de la Sota

**Australia**

Roger Byard

Lesley Hawson

Roy Kirkwood

John Pluske

**Austria**

Christine Aurich

Durga Chapagain

Marc Drillich

Martina Floeck

Nikola Katic

Daniela Klein-Jöbstl

Akos Pakozdy

**Belgium**

Filip Boyen

Ginette Deby-Dupont

Philippe Delahaut

Jeroen Dewulf

Axel Mauroy

Luc C Poncelet

Jérôme Ponthier

Emmelie Stock

Eric Thys

Lieven Vlaminck

**Brazil**

David Driemeier

Vinicius Machado

Denise Otsuki

Alexandre Souza

**Canada**

Burim Ametaj

Paul De La Bastide

Sagi Denenberg

Rob Foster

Robert Friendship

Emma Read

Cheryl Waldner

Scott Weese

**China**

Ineke Rood

**Czech Republic**

Pavel Alexa

**Denmark**

Jens Frederik Agger

Peter Ahrens

Lis Alban

Lars Andresen

Øystein Angen

Betina Børresen

Janne Winther Christensen

Jan Dahl

Peter Damborg

Hanne Gredal

Carsten Grøndahl

Tariq Halasa

Mette Herskin

Charlotte Hjulsager

Hans Houe

Henrik Elvang Jensen

Trine Jensen

Sven Erik Jorsal

Gregers Jungersen

Jesper Krog

Lars Larsen

Páll S Leifsson

Peter Lind

Liza Nielsen

Søren Saxmose Nielsen

John Olsen

Aage Kristian Olsen

Lisbeth Olsen

Tina Pihl

Inge Tarnow

Anne-Helene Tauson

**Estonia**

Brian Lassen

Kerli Raaperi

**Finland**

Magnus Carl Andersson

Maria Fredriksson-Ahomaa

Laura Hanninen

Marja-Liisa Hänninen

Mari Heinonen

Anna Hielm-Björkman

Sami Junnikkala

Liisa Kaartinen

Maria Kareskoski

Anna-Mariam Kiviranta

Ulla-Maija Kokkonen

Outi Laitinen-Vapaavuori

Ann-Katrin Llarena

Leena Maunula

Camilla Munsterhjelm

Antti Oksanen

Claudio Oliviero

Sinikka Pelkonen

Olli Peltoniemi

Satu Pyörälä

Marja Raekallio

Merja Rantala

Mirja Ruohoniemi

Heli Simojoki

Thomas Spillmann

Juhani Taponen

Suvi Taponen

Juha Tuukkanen

Anna Elisabet Valros

**France**

Armelle Diquélou

Sylvie Nazaret

Luca Zilberstein

**Germany**

Ralf Dittrich

Andrea Feßler

Wolfgang Heuwieser

Marion Hewicker-Trautwein

Martina Hoedemaker

Korinna Huber

Hana Hünigen

John Ilukor

Cornelia Kraus

Arno Lindner

Ralf Mueller

Kerstin Müller

Marion Piechotta

Katharina Schaufler

Veronika Stein

Gamal Wareth

**Greece**

Leonidas Leontides

**Hungary**

Szabolcs Nagy

Pál Rafai

**Iceland**

Sigridur Bjornsdottir

Vala Friðriksdóttir

Jonmundsson Jon Vidar

**India**

Praveen Malik

**Ireland**

Nicola Kelly

Luke O’Grady

**Israel**

Nahum Shpigel

**Italy**

Moreno Bondi

Paolo Bosi

Marta Brscic

Antonio Carminato

Fabrizio Ceciliani

Fabio De Rensis

N Decaro

Arcangelo Gentile

Giulio Grandi

Domenico Iannelli

Cecilia Luvoni

Saverio Paltrinieri

Algimantas Paulauskas

Elisa Pieragostini

Erminio Trevisi

**Japan**

K Harada

Naoaki Matsuki

Kurita Tadayoshi

Han Sang Yoo

**Netherlands**

Birgitta Duim

Menno Holzhauer

Henk Smits

Nanda Ursinus

Els Van Coillie

Arie Van Nes

Cornélie Westermann

**New Zealand**

Jon Hickford

**Nigeria**

Henry Kiara

**Norway**

Ole Herman Ambur

Hege Brun-Hansen

Carlos Das Neves

Anna Vigdis Eggertsdottir

Cecilie Ersdal

Wenche Farstad

Karin Hultin Jäderlund

Carl Fredrik Ihler

Terje Josefsen

Gunnar Klemetsdal

Thor Landsverk

Lars Moe

Ane Nødtvedt

Madelaine Norström

Ingebjørg Nymo

Oivind Oines

Kristian Prydz

Birgit Ranheim

Lucy Robertson

Maria Stokstad

Marianne Sunde

Arnfinn Sundsfjord

Stein Thoresen

Martha Jakobsen Ulvund

Mette Valheim

Helene Wisloff

**Poland**

Piotr Jurka

Agata Miska-Schramm

Wojtek Nizanski

Kamil Torres

**Portugal**

David Ferreira

A.P. Lopes

Rita Payan Carreira

**Slovakia**

Peter Massanyi

**Slovenia**

Martina Klinkon

**South Africa**

Gideon Brückner

Aspinas Chapwanya

BB Dogonyaro

**Spain**

Angel Abuelo

Javier Dieguez-Uribeondo

Ana García-Pérez

Ignacio Moriyon

**Sweden**

Göran Andersson

Eva Axner

Viveca Båverud

Bjorn Bengtsson

Anna Bergh

Annika Bergström

Kerstin Bergvall

Björn Ekesten

Ulf Emanuelson

W. Freddy Fikse

Caroline Fossum

Pia Funkquist

Ulrika Grönlund

Pia Gustås

Sara Hägglaund

Ragnvi Hagman

Jeanette Hanson

Kerstin Hansson

Eva Hellmen

Gete Hestvik

Odd Viking Höglund

Magdalena Jacobson

Bernt Jones

Lulia Karlsson

Charles Ley

Inger Lilliehöök

Ronny Lindberg

Ulf Magnusson

Ann-Kristin Nyman

Elisabeth Persson

Ylva Persson

Karin Persson Waller

Henrik Rönnberg

Bodil Strom Holst

Anna Tidholm

Jonas Waldenström

**Switzerland**

Ueli Braun

Josef Gross

Sonja Hartnack

Annette Nigsch

Reto Zanoni

**United Republic of Tanzania**

Mark Rweyemamu

**Thailand**

Paweena Thuwanut

**Turkey**

Enver Beytut

**United Kingdom**

Briony Alderson

David Bennett

Alison Cody

Janet Daly

Andy Durham

Sue Dyson

Kiterie Faller

OP Forman

Simon Graham

Javier Guitian

Danielle Gunn-Moore

Clare Hamilton

Gareth Hateley

Paul Macfarlane

Elspeth Milne

Siobhan Mullan

Jasmin Paris

Adrian Philbey

Haylay Randle

Scott Reid

Phil Scott

Gert Ter Haar

L.A. Withcomb

James W Yeates

**United States of America**

Andrew Bowman

Munashe Chigerwe

Andrés Contreras

Michael Davis

Melissa Elischer

James Ferguson

Cara Field

Lisa Freeman

Laura Garrett

H. Carl Gelhaus

Laurie Goodrich

T Grandin

Kimberly Greer

Sanjeev Gumber

H. Hilton

Kathryn Holcomb

Karsten Hueffer

Cheri Johnson

Michelle Kutzler

Emily Lankau

Gwendolyn Levine

Christoph Mans

Kyle Mathews

John Mcnamara

Emily Miller-Cushon

T. G. Nagaraja

Nathan Nelson

Anita Oberbauer

EA Ostrander

Robert Paine

SR Platt

Sarah Ralston

Sanjeewa Ranathunga

Karen Register

Allen Roussel

Helena Rylander

Rebecca S Sayre

Sandra Scholes

Andy Shores

Nathan Slovis

Geof Smith

David Steffen

George M Strain

WB Thomas

Katherine Tolbert

Susan Tornquist

